# An Overview on Recent Advances in Biomimetic Sensors for the Detection of Perfluoroalkyl Substances

**DOI:** 10.3390/s24010130

**Published:** 2023-12-26

**Authors:** Fatemeh Ahmadi Tabar, Joseph W. Lowdon, Soroush Bakhshi Sichani, Mehran Khorshid, Thomas J. Cleij, Hanne Diliën, Kasper Eersels, Patrick Wagner, Bart van Grinsven

**Affiliations:** 1 Laboratory for Soft Matter and Biophysics ZMB, Department of Physics and Astronomy, KU Leuven, Celestijnenlaan 200 D, B-3001 Leuven, Belgium; fatemeh.ahmaditabar@kuleuven.be (F.A.T.); soroush.bakhshisichani@kuleuven.be (S.B.S.); mehran.khorshid@kuleuven.be (M.K.); 2 Sensor Engineering Department, Faculty of Science and Engineering, Maastricht University, P.O. Box 616, 6200 MD Maastricht, The Netherlandsthomas.cleij@maastrichtuniversity.nl (T.J.C.); kasper.eersels@maastrichtuniversity.nl (K.E.); bart.vangrinsven@maastrichtuniversity.nl (B.v.G.)

**Keywords:** molecularly imprinted polymers, biomimetic sensors, polyfluoroalkyl substances, environmental pollution, aptamers

## Abstract

Per- and polyfluoroalkyl substances (PFAS) are a class of materials that have been widely used in the industrial production of a wide range of products. After decades of bioaccumulation in the environment, research has demonstrated that these compounds are toxic and potentially carcinogenic. Therefore, it is essential to map the extent of the problem to be able to remediate it properly in the next few decades. Current state-of-the-art detection platforms, however, are lab based and therefore too expensive and time-consuming for routine screening. Traditional biosensor tests based on, e.g., lateral flow assays may struggle with the low regulatory levels of PFAS (ng/mL), the complexity of environmental matrices and the presence of coexisting chemicals. Therefore, a lot of research effort has been directed towards the development of biomimetic receptors and their implementation into handheld, low-cost sensors. Numerous research groups have developed PFAS sensors based on molecularly imprinted polymers (MIPs), metal–organic frameworks (MOFs) or aptamers. In order to transform these research efforts into tangible devices and implement them into environmental applications, it is necessary to provide an overview of these research efforts. This review aims to provide this overview and critically compare several technologies to each other to provide a recommendation for the direction of future research efforts focused on the development of the next generation of biomimetic PFAS sensors.

## 1. Introduction

**Figure 1:**
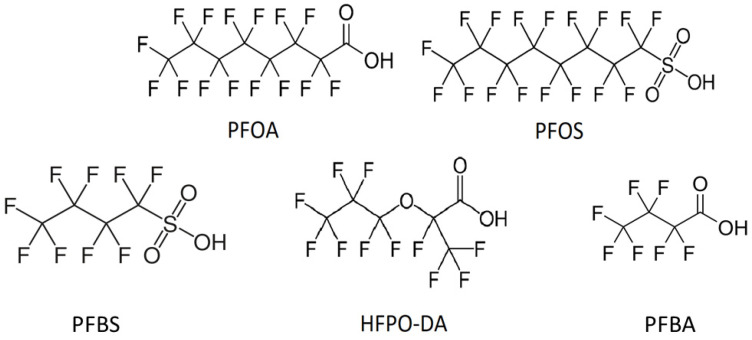
The chemical structures of perfluorooctanoic acid PFOA (molecular weight MW: 414 g/mol), perfluorooctane sulfonate PFOS (MW: 500 g/mol), perfluorobutanesulfonic acid PFBS (MW: 300 g/mol), hexafluoropropylene oxide dimer acid HFPO-DA (MW: 330 g/mol), and perfluorobutanoic acid PFBA (MW: 214 g/mol).

Polyfluoroalkyl substances (PFAS) are a category of organic molecules consisting of a fully fluorinated alkyl chain [1]. Perfluorooctanoic acid (PFOA), perfluorooctane sulfonate (PFOS), and hexafluoropropylene oxide dimer acid (HFPO-DA)—see Figure 1—are some of the most common and problematic PFAS with widespread use in different products such as semi-conductors, firefighting foams, lubricants, and non-stick coatings [2,3,4]. As they have been extensively used, they can be found in surface/drinking water and sediments [5,6]. Moreover, these compounds are capable of bioaccumulation in human and animal tissue, have high chemical and thermal stability, and are potentially carcinogenic and neurotoxic [7,8,9]. This has raised concerns and debates about the potential risks of PFAS species and forced legislative bodies to react [10,11,12]. For instance, in 2020, the European Commission (EC) decided to reframe the EU Drinking Water Directive and incorporated a maximum health advisory level of 0.1 µg/L for each individual type of PFAS molecules [13]. There are also short-chain PFAS molecules with only four carbon atoms such as perfluorobutanesulfonic acid (PFBS) and perfluorobutanoic acid (PFBA) that are considered as toxic; however, their serum half-life inside the human body ranges from a few days to less than a month [14]. This is considerably shorter than for PFOS, with an estimated half-life between 3.4 and 5 years; as a consequence, the bioaccumulation seems less critical.

Currently, PFOA detection typically relies on liquid chromatography paired with mass spectrometry [15,16,17]. Although this method is highly sensitive and selective, it usually requires elaborate sample preparation, expensive equipment, and well-trained personnel. As a result, it is not suitable for a fast and facile examination in routine environmental monitoring [18,19,20]. Consequently, it is highly desirable to have sensors available that can provide a facile, sensitive, selective, fast, quantitative, and cost-effective way of detecting PFOA directly in the field. User-friendly bio (mimetic) sensors can meet these demands.

In general, bio- and chemosensors consist of two main components, the receptor layer and the transducer [21,22]. The receptor layer recognizes the target based on specific molecular interactions and is combined with a transducer, which converts the binding events between targets and receptors into interpretable data [23]. The receptor choice has a direct impact on the detection range and selectivity of the sensor. Therefore, the this review will focus on the different receptor types that have been developed for the detection of PFAS molecules. To date, biomimetic receptors such as molecularly imprinted polymers (MIPs), aptamers, and metal–organic frameworks (MOFs) have been synthesized for PFAS detection. The aim is to eventually integrate these receptors into portable devices for the onsite detection of these compounds [24]. Recently, P450-type enzymes were also identified that can biodegrade PFAS molecules, which may offer potential for enzymatic PFAS detection in future [25,26].

MIPs are polymeric matrices with predetermined recognition properties for a certain molecule or a set of similar molecules [27,28]. The applications of MIPs initially focused on separation and extraction processes [29]. In recent years, MIPs have also been applied in a wider context, including solid-phase extraction, drug delivery, catalysis, and environmental and chemical sensing [30,31,32,33]. A key benefit of MIP technology is that MIP-based sensors can be developed for a wide variety of targets [34,35,36]. This includes environmental contaminants, chemical and biological compounds, as well as industrial chemicals [35,37,38]. Regarding specifically fluorinated contaminants, we refer the reader to [39,40,41,42,43]. 

The specific binding interaction of MIPs with their targets leads to changes in physical characteristics including mass, electrochemical impedance, thermal resistance, and fluorescence, which can be used as transducer mechanisms in the sensor [44,45,46,47,48]. In comparison to biological receptors such as antibodies and enzymes, MIPs are stable over a wide pH and temperature range, while their sensitivity and selectivity towards their target are only marginally lower. They are relatively straightforward and low-cost to prepare with adjustable surface properties and applicability outside a laboratory environment [49,50,51]. While MIPs are chemically and physically more stable than natural receptors, there are still points of attention. First of all, incomplete extraction of template molecules can result in template leakage during the measurements. Secondly, the recognition properties of the binding sites can be heterogeneous, which may affect the reproducibility of analytical results [52]. These disadvantages can be overcome by a better control over the polymerization process for example by using nanoMIPs [53,54,55].

Aptamers are receptors based on nucleic acids (DNA or RNA) that bind a particular target analyte, or a group of target analytes, by folding into specific conformations, van der Waals and electrostatic interactions, and/or hydrogen bonds [56]. Aptamers can be obtained by a combinatorial selection process known as systematic evolution of ligands by exponential enrichment SELEX [57]. SELEX involves the progressive selection of oligonucleotide sequences from a large pool of randomly generated sequences towards high binding affinities between the desired target and oligonucleotide sequences. Aptamers typically contain 25–80 bases, which will fold into complex tertiary structures. Aptamers can be designed to recognize various analytes with high affinity and specificity, including organic dyes, toxins, and proteins [58]. Furthermore, it is possible to select aptamers in a way that they recognize only an individual target analyte, or to bind a set of analytes with a similar structure. Thanks to their small size, it is possible to incorporate different aptamers into the same sensor to detect multiple chemicals in parallel, with each aptamer selective towards a different analyte. Similar to MIPs, aptamers can also be combined with various transducer principles [59]. Unfortunately, aptamer development for a new target can be expensive and time consuming, and it is hard to scale up their synthesis to mass production. In comparison to MIPs, aptamers have less physical and chemical stability towards harsh environmental conditions, and they are prone to enzymatic degradation [60].

Metal–organic frameworks (MOFs) are porous materials consisting of rigid inorganic groups and flexible organic linker ligands. MOFs are potential candidates for selective chemical sensing with low detection limits owing to their extremely high surface area, variability of metal nodes, and modifiable organic linkers to provide adjustable binding sites [61,62]. By choosing different metal clusters for the organic linkers to coordinate around, surface characteristics and pore sizes can be adjusted [63]. Due to their stability, they can be used repeatedly to detect specific analytes [64]. MOFs with different metal centers can trap PFAS by strong affinity interactions, making them promising for the development of PFAS-detection platforms [65]. MOFs can furthermore be combined with MIPs to improve their sensing properties [66,67,68], while also the combination of MOFs with aptamers has been reported in the literature [69]. For strategies to combine molecular imprinting with aptamer technology, we refer to the recent review article by Zhou and coworkers [70]. 

The aim of this review is to present a better understanding of the advantages that each of these receptors (MIPs, aptamers, and MOFs) offers for PFAS determination and how to optimize the design of a sensor layer in view of the desired application. We therefore aim at providing an overview of the most recent innovations in biomimetic PFAS detection. Opportunities for each receptor type will be analyzed and compared to potential obstacles and challenges that sensors based on these type of receptors will face when implementing them into a real-life application. This way, this literature overview seeks to provide recommendations for future research towards the development of PFAS sensors for direct application in environmental screening.

## 2. PFAS Sensing with Receptors Made via Imprinting Technology

**Figure 2:**
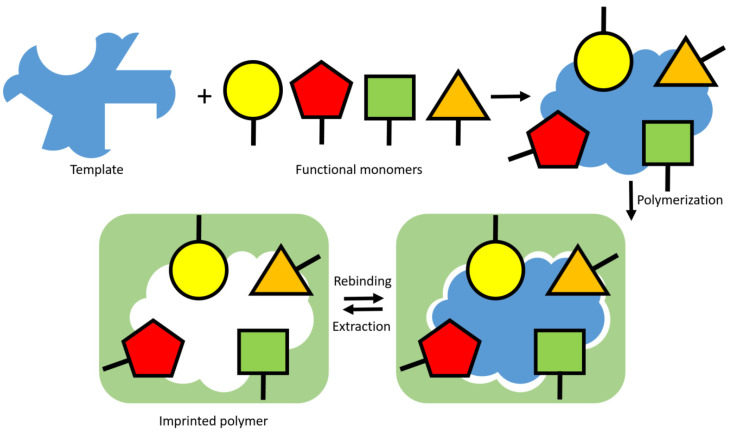
Schematic representation of the concept of molecular imprinting. The synthetic receptor formation starts by a stereochemical arrangement of functional monomers around a template of interest through self-assembly. The most adopted approach consist of adding an initiator and crosslinker to the pre-polymerization mixture and thermally or UV-induced polymerization. This creates a highly crosslinked polymeric network around the template that serves as a plastic mold to which the template can specifically rebind upon extraction.

MIPs are synthetic polymer structures prepared by various polymerization methods between a crosslinker, template and one or more functional monomers in a porogenic solvent [71,72]. During the polymerization, specific interactions take place between the template and the monomer’s functional groups [73]. Subsequently, when the template is removed with an appropriate solvent, molecular cavities are created whose shape, size, structure and functionality are complementary to the template analyte and are able to detect the target in another matrix through a “lock–key interaction”; see Figure 2 [74,75,76]. The sensitivity and selectivity of the resulting sensing tool strongly depend on the affinity of the imprinted polymer for the target [77]. 

Optimizing the stoichiometric ratios between the crosslinker, monomer, and template will improve this affinity and the binding capacity of the MIP [78]. The sensing capability of a MIP is usually compared with a non-imprinted polymer (NIP) that is synthesized in an identical manner, but without the presence of the template analyte to evaluate the effect of imprinting [79]. Clearly, the major challenge is to achieve MIPs with a high affinity and specificity for the target analyte [77].

Most approaches to synthesize MIPs are focused on free radical polymerization as this method offers a facile and low-cost route for creating large batches of MIPs. However, these particles are highly heterogeneous which makes it hard to create reproducible sensors. Therefore, more controllable methods of creating homogenous, high affinity MIPs have been developed by more controllable methods such as suspension, precipitation and emulsion polymerization [80]. However, all these methods will result in the creation of MIP particles that still need to be deposited on a planar sensing electrode. Therefore, methods to directly deposit MIPs onto a conductive sensing surface, such as electropolymerization, are becoming increasingly popular [81,82]. The polymerization method is of high importance as it affects the size, shape, homogeneity, thermal durability, and binding capacity of the resulting MIP particles or layers [83,84,85,86].

**Figure 3:**
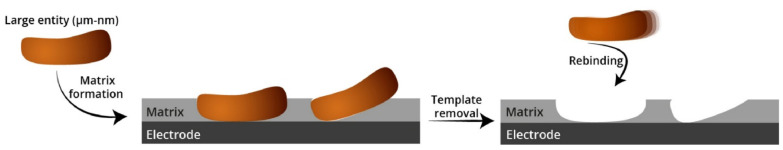
Schematic illustration of molecular imprinting directly on an electrode surface. As shown, the templates leave the imprinted cavities on the top of polymeric matrix and target analytes can rebind with the imprints. Figure reprinted from [95]. Copyright 2022, with permission from Elsevier.

MIPs can also be synthesized directly on the sensor surface by depositing a thin polymer layer, which is imprinted with the target [55,87,88]. With this method, the binding cavities are mostly located at the outer layer of the substrates; see Figure 3 [89,90]. Surface imprinting can be accomplished directly on the electrode’s surface, or the outer layer of a carrier such as nanoparticles and nanofibers. The MIP layer usually embeds only part of the template, which can be sufficient for the selective rebinding of the template after its removal. To date, there are several studies reporting on the use of surface-imprinted polymers for PFAS sensing [91,92,93,94].

The synthesis approach depends on the specific application that is targeted. In some cases, it is preferable to prepare polymers separately by for instance bulk or suspension polymerization to facilitate quality control, enable mass production, and control surface coverage. Nevertheless, these methods can have drawbacks including difficulty in controlling the layer thickness, embedding of the template, and cavity accessibility [96,97]. It is worth mentioning that one of the main challenges in conventional polymerization methods is the incorporation of polymerized MIPs into the sensors and imprinting of polymers on the electrode’s surface is a feasible solution to this issue [98]. Molecular imprinting, directly on the electrode’s surface, offers several benefits such as binding sites with better accessibility, faster binding kinetics, and faster mass transport [52]. In general, larger macromolecular entities (cells, proteins, bacteria, etc.) cannot be dissolved in a pre-polymerization mixture and therefore need to be imprinted on the surface of a solid substrate. In addition to the polymerization method, the selection of monomers will affect the performance of MIPs and depending on the monomers; the MIPs will function best in a specific pH range [99].

### 2.1. Imprinting with Conventional Polymerization Methods

Bulk free radical polymerization is the most common route to prepare MIPs for low-molecular-weight compounds such as PFAS, owing to its simplicity and low production costs for large amounts [100]. Once the polymerization is completed, the product is a solid polymeric structure that needs to be crushed, ground and then eluted with solvents. The synthesized product can be sieved with an appropriate mesh size to obtain particle sizes adapted to a particular application, which may vary from micrometer to submicrometer diameters [101,102]. A disadvantage is the grinding and sieving process, which may take long and result in significant product waste [43,103].

**Figure 4:**
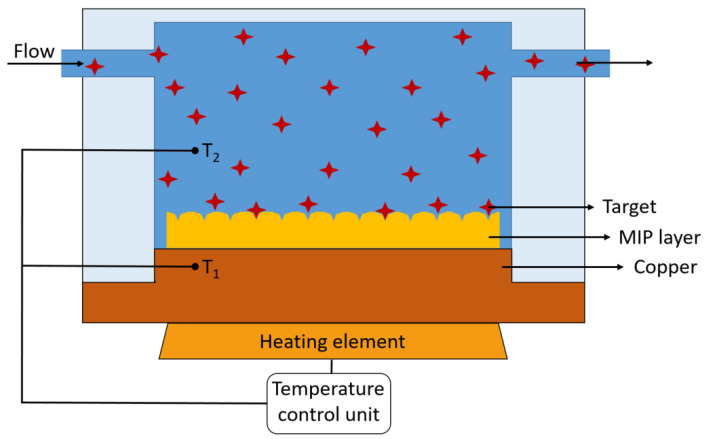
Schematic illustration of the heat transfer method setup. The temperature T_1_ is constant by using a heating element and a temperature control unit. The temperature in the fluid (T_2_) is varying by the changes on the MIPs layer.

**Figure 5:**
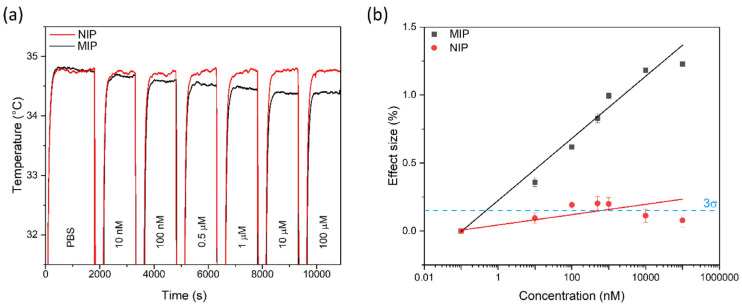
(**a**) Temperature response (T_2_) for both MIP and NIP after exposure to different concentrations of PFOA in PBS (T_1_ was kept constant at 37 °C). (**b**) Dose–response curve of MIP- and NIP-covered sensor chips obtained by HTM. The LoD for these measurements was calculated as 0.48 nM based on the intercept of the 3σ line with the MIP curve. Reproduced with permission from [104]. Copyright 2023, Elsevier (CC-BY).

In 2023, Ahmadi Tabar et al. synthesized PFOA MIPs by bulk free radical polymerization and optimized the receptor by changing the molar ratio of the polymerization components (1/4/12 for template/monomer/crosslinker) to maximize the target affinity and selectivity [104]. Rebinding of PFOA to the MIPs was assessed by a thermal transducer known as heat transfer method HTM [105,106]. This method works by recording the thermal resistance between the chip and the sample with two temperature sensors while the chip is covered with a MIP layer. The temperature below the chip (T_1_) is kept constant at, e.g., 37 °C using a temperature control unit, and the output temperature (T_2_, above the MIPs layer) is measured; see Figure 4. Increasing the concentration of PFOA led to a concentration-dependent decrease in T_2_ for the MIP chip, while temperature changes were negligible for its NIP counterpart; see Figure 5a. The effect size (%) was calculated by dividing the temperature changes by the initial temperature (°C), which is plotted in function of the PFOA concentration in Figure 5b. Increasing T_1_ from 37 °C to 40 °C minimized the noise on the signal and lowered the detection limit LoD from 0.48 nM to 22 pM. This LoD is below the PFOA contamination level (0.1 µg/L: 0.24 nM) stated in the EU Drinking Water Directive [13].

The results also demonstrated that the sensor is selective with the cross selectivity below 30% for other PFAS molecules such as heptafluorobutyric acid (HFBA), and perfluorobutanesulfonic acid (PFBS). Furthermore, the sensor was able to detect PFOA in spiked environmental samples including river water and soil in the regulatorily relevant concentrations with LoD values of 91 and 154 pM, respectively. These results provided proof of the potential application of MIP-based sensors in routinely monitoring of environmental samples for PFAS contamination. The benefit of combining bulk MIPs with a thermal readout principle is that the synthesis approach is scalable and rebinding results in an easily interpretable increase in the thermal resistance, respectively a decrease in the temperature T_2_.

Precipitation and emulsion polymerization are other techniques for synthesizing MIPs [107,108,109]. In these methodologies, the polymerization approach is the same as bulk polymerization, but the post-processing stages are not necessary, resulting in fewer steps and, more crucially, a lower risk of damaging the binding sites. The emulsion polymerization is rapid and has a mechanical dispersion system constantly working in the presence of a surfactant, and it can achieve continuous production [110]. There is no need for a surfactant for precipitation polymerization and, during this polymerization, the growing polymer segregates from the solution, finally forming MIP particles with micro or submicrometer dimensions [111,112].

Cao and coworkers prepared MIPs for the selective adsorption of PFOA in aqueous solutions by precipitation polymerization of acrylamide in the presence of PFOA as the template molecule [39]. The concentration of PFOA in Milli-Q water was measured by liquid chromatography with tandem mass spectrometry. The optimized MIPs showed a high affinity for PFOA, and the uptake percentage by the MIPs was 1.3–2.5-fold higher than that of the NIP when exposed to PFOA alone. The MIPs adsorbent showed a high selectivity for PFOA over other PFAS molecules such as PFOS and perfluorodecanoic acid. Furthermore, the reusability of the MIPs adsorbent was confirmed in five consecutive adsorption–desorption cycles without a notable decrease in the PFOA uptake. In summary, the results were promising in terms of selectivity, the sorption capacity of the resulting MIPs and the relatively low batch-to-batch variability, which also makes the MIPs promising candidates for PFOA detection. In this context, it is noteworthy that there is also literature on PFAS absorption using MIPs [43].

### 2.2. Imprinting by Electropolymerization

Surface imprinting of low-molecular-weight compounds can be achieved by depositing and imprinting a polymer layer directly on an electrode surface via electropolymerization, which is a particularly useful method in combination with electrochemical transducers [113,114]. By cycling the potential in a predetermined range with a given sweep rate, the electroactive monomer (such as aniline, o-phenylenediamine, and pyrrole) will be electropolymerized and the substrates will be coated by a very thin layer of polymer [81,92,115]. In this method, the potential range, the sweep rate and the composition of pre-polymerization mixture can control and optimize the adherence and morphology of the imprinted polymer layer on the electrode [116]. The main advantage of this method is that the polymer layer thickness is controllable in a reproducible manner with low batch-to-batch variability. In addition, it is possible to automate the process. Finally, it is feasible to obtain better binding capacity and sensitivity, which is the result of thinner and more homogenous layers. Additionally, because of the ultrathin MIPs film that is produced and the proximity of the imprinted cavities to the surface, this approach makes template removal facile. Therefore, electropolymerization is typically a straightforward and fast method that involves these phases: dissolution and interaction of an electroactive monomer with the given template in a solvent (the solvent can even be water with electrolytes), coating electrochemically, and finally the elution of the templates [30].

**Figure 6:**
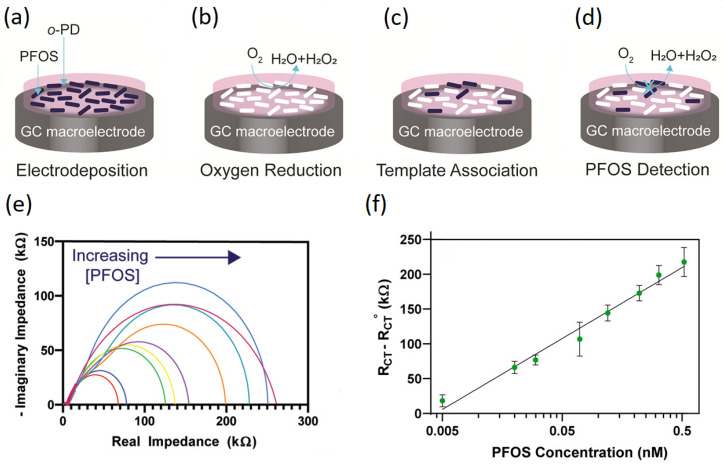
Schematic illustration of the PFOS detection procedure. (**a**) Electropolymerization of o-phenylenediamine (o-PD) on a glassy carbon macroelectrode. PFOS molecules are shown as black ovals and the white ovals are the biding cavities remained after PFOS removal. (**b**) Driving of oxygen reduction on the MIP-modified electrode. (**c**) Rebinding of the template molecule with the MIPs. (**d**) Blocking the electrochemical signal of the redox reaction by bound PFOS molecules. (**e**) The R_ct_ values increases with the increase in the PFOS concentration. (**f**) The normalized R_ct_ against the logarithm of PFOS concentration. Reproduced with permission from [93]. Copyright 2020, American Chemical Society (CC-BY).

Clark et al. performed electropolymerization on a glassy carbon electrode surface by cyclic voltammetry in an aqueous solution containing o-phenylenediamine (o-PD) and PFOS [93]. Figure 6 shows a schematic illustration of fabrication process for the MIP-based sensing platform. After template removal by a water/methanol solution, they performed oxygen reduction (O_2_ was dissolved in water) on the electrode, as illustrated in Figure 6b. In differential pulse voltammetry (DPV), the electrode revealed oxygen reduction peaks around −0.5 and −0.9 V and the first one was used as an electrochemical signal to plot the calibration curves. PFOS was able to associate with the MIPs (Figure 6c) and block the electrochemical signal of the oxygen redox reaction (Figure 6d). In this electrochemical spectroscopy technique, as the PFOS concentration increased, the effective electrode surface area decreased. This can be seen by the increase in the charge-transfer resistance (R_ct_), which is the diameter of the semi-circle of the Nyquist plots in Figure 6e. The curve in Figure 6f is the change in R_ct_ with respect to the baseline against the logarithm of PFOS concentration. Furthermore, the sensor achieved a detection limit of 3.4 pM for PFOS using electrochemical impedance spectroscopy without a redox mediator such as ferrocene carboxylic acid. Clark et al. also revealed that two common environmental interferents (sodium chloride and humic acid) do not affect the sensor signal. Moreover, it was feasible to obtain reproducible results with matrices such as river water using impedance spectroscopy.

In a similar approach, Karimian and co-workers developed an electrochemical sensing platform for the determination of trace amounts of PFOS in water [94]. The sensor consisted of a gold electrode functionalized with a thin layer of MIPs, synthesized by electropolymerization of o-PD with PFOS as the template. The sensor was activated by template elution with adequate solvents. Ferrocene carboxylic acid (FcCOOH) was used as a redox probe, capable of generating analytically useful voltametric signals by competing for the recognition regions with PFOS, while PFOS itself is not electrochemically active. According to the observations, the voltametric signal at the MIP-coated electrode decreased gradually when the sensor was submerged in PFOS containing samples in deionized water, scaling inversely with the PFOS concentration. According to the selectivity results, other PFAS molecules including PFOA, HFBA, and PFBS caused maximally 20% change in the signal normalized to PFOS. The sensor also demonstrated a limit of detection of 0.04 nM, and an acceptable reproducibility and repeatability.

### 2.3. Imprinting on Nanoparticles

In order for MIPs to work optimally, the number of binding cavities is essential. One strategy to increase the number of binding sites can be achieved by increasing the thickness of the imprinted polymer layer. However, this will reduce diffusion and therefore mass transport and interaction with the transducer [117,118]. One practical solution is to synthesize MIPs on the surface or the external layer of a particular carrier with a large surface area [96]. This improves elution and rebinding of template- and target molecules, it decreases the amount of non-accessible binding sites. In addition, it enhances both the availability of the target to the binding regions as well as the corresponding kinetics [118]. Polystyrene microspheres, silica nanoparticles, carbonaceous nanomaterials, and magnetic nanoparticles are examples of frequently employed carriers [119,120,121,122]. The surfaces of imprinted substances are controllable, and the recognition regions with high density are easily accessible by the targets, improving the adsorption capacity and effectiveness [118,123,124].

**Figure 7:**
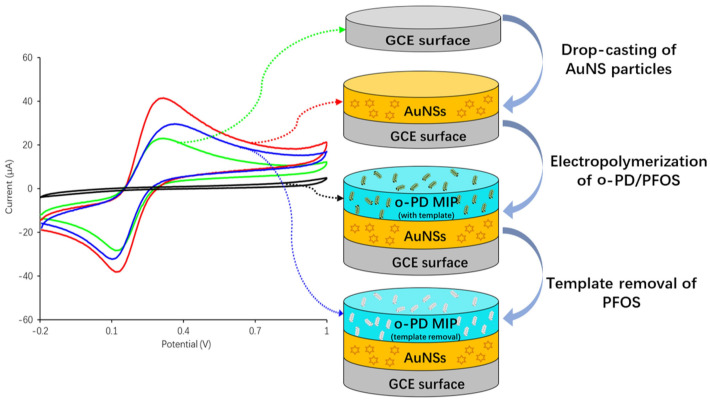
Schematic illustration of the voltametric sensor consisting of MIP and gold nanostars (AuNS) coatings for PFOS determination. Right: The GCE surface is first modified with AuNS and then electropolymerized with o-PD using cyclic voltammetry (CV). Left: The CV curve and the probes’ oxidation peak for pristine GCE, AuNS/GCE, and MIP/AuNS/GCE before and after PFOS removal. Figure adapted from [125]. Copyright 2021, with permission from Elsevier.

In 2022, Lu et al. modified pristine glassy carbon electrodes (GCE) with a thin layer of gold nanostars (AuNS) by drop-casting to increase the sensitivity of the electrode to the electrochemical probe (ferrocene carboxylic acid) [125]. These AuNs-coated GCEs were then coated with a layer of PFOS-imprinted o-PD using cyclic voltammetry for electropolymerization to enhance the sensitivity towards PFOS; see Figure 7. The interaction between the MIP layer and PFOS was analyzed by the oxidation peak of FcCOOH (Fe^2+^/Fe^3+^) using DPV. Figure 7 (left) indicates that the oxidation peak has entirely disappeared for the MIP/AuNS/GCE before PFOS removal. This means that the MIP layer is able to completely block the charge transfer between the working electrode and the solution. This voltametric sensor was able to detect PFOS with a limit of detection (LoD) of 0.015 nM calculated by using the 3σ method. The suggested sensing platform was also capable of detecting trace levels of PFOS in tap water. However, during the measurements significant interferences with perfluorobutanoic acid (PFBA) or PFBS were observed. This observation can be explained, by the smaller sizes of PFBA and PFBS molecules, enabling them to pass across the MIPs layer and occupy the PFOS-shaped cavities by non-specific binding. Therefore, it is required to first screen an unknown sample for the presence of small PFAS molecules such as PFBA and PFBS.

Gao et al. prepared an electrochemical sensor for the detection of PFOS in real water samples [126]. The sensor (PFOS-MIPPDA/AuNPs/GCE) was made from a GCE modified with gold nanoparticles (AuNPs) and an electropolymerized molecularly imprinted polydopamine (DA) coating with PFOS as the template. PFOS detection was achieved by using DPV and K_3_[Fe(CN)_6_] as the detection probe. The results revealed that the developed PFOS-MIPPDA/AuNPs/GCE sensor was able to determine PFOS with a nanomolar detection limit. The sensor also showed promising results for analyzing real water samples including tap, lake, and canal water.

Zheng et al. developed a photoluminescence sensor (PL) for selective detection and quantification of PFOA based on MIP-coated CdTe@CdS quantum dots (QDs) [127]. This optical sensor provided fast and sensitive detection of PFOA in the presence of common interferents by the PL quenching via target rebinding into the recognition cavities in the polymeric layer. Furthermore, the fabricated sensor demonstrated a good linearity in the range from 0.25 to 15.00 µmol/L with a PFOA detection limit of 25 nM.

### 2.4. Imprinting on Nanofibers

**Figure 8:**
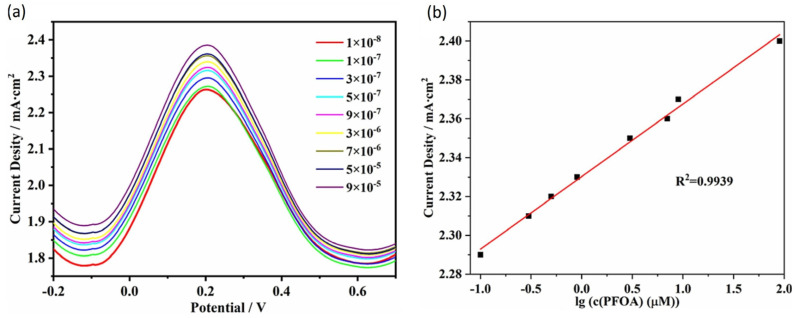
(**a**) Differential pulse voltammetry (DPV) of MIPs Co/Fe@CNF at different PFOA concentrations in molar units and (**b**) linear relationship between the current density and the logarithm of the PFOA concentration. Figure adapted from [67]. Copyright 2023, with permission from Elsevier.

As mentioned above, one solution to improve the recognition performance of the sensor is to synthesize MIPs on the exterior layer of a particular carrier with a high surface-to-volume ratio. Electrospun fibers can be considered as promising carriers because of their large surface area. Wang et al. successfully prepared a MIPs MOFs (Co/Fe)-driven carbon nanofiber (Co/Fe@CNF) electrode for electrochemical determination of PFOA, more information on MoFs is provided in Section 3.2 [67]. MIPs were formed by electropolymerization of pyrrole with PFOA as template. Owing to the strong adsorption force between the imprinting sites of MIPs and PFOA, PFOA molecules could reach the surface of electrode. In DPV measurements, the peak at 0.2 V (corresponding to PFOA) was used to plot the calibration curves (Figure 8a). The response current of MIPs Co/Fe@CNF electrode increased with the increase in PFOA concentration (Figure 8b). Under optimum conditions, the resultant MIPs Co/Fe@CNF was able to determine PFOA with a linear response with respect to the logarithm of the PFOA concentration and the limit of detection was 1.07 nM. The as-developed sensor also worked properly for measuring PFOA in real wastewater samples and it was a promising candidate for the determination of PFOA in environmental water samples.

In recent literature, there are many more PFAS sensors based on MIPs and Table 1 provides a comparative overview on the polymer composition, the readout principle, the limit of detection, and the sample type, which has been used for the measurements.

## 3. PFAS Sensing with Other Synthetic Receptors

### 3.1. PFAS Sensing with Aptamers

**Figure 9:**
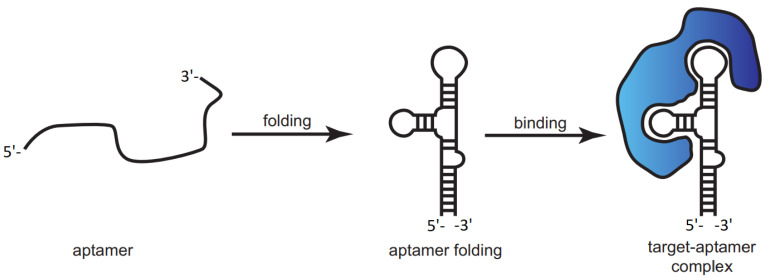
Schematically representing the formation of a target–aptamer complex. The aptamer folds into a 3D structure, upon which it binds to the target molecule. Reproduced with permission from [132]. Copyright 2017, Society for Neuroscience (CC-BY).

As mentioned before, aptamer molecules undergo conformation changes in the presence of various target analytes and bind to them with high selectivity and affinity [129,130]. They are gaining increasing attention from researchers as a substitute to antibodies as specific elements for target molecule recognition owing to their flexibility, relatively small size, and easy chemical modification. Figure 9 indicates how the aptamer goes through conformational changes in the presence of a target molecule. Different kinds of reactions and physical factors can participate in the formation of aptamer-target complexes, namely hydrogen bonding, polar groups, shape complementarity, and van der Waals forces [58]. Aptasensors are a class of biosensors that combine a synthetic, biomimetic recognition element (aptamer) with chemical/physical transduction for precise detection of various target molecules. These sensors can potentially be used in environmental monitoring due to their high sensitivity and selectivity, high efficiency, and the ability to miniaturize these platforms [131]. Therefore, aptasensors can be developed for screening of PFAS and other existing pollutants in water.

**Figure 10:**
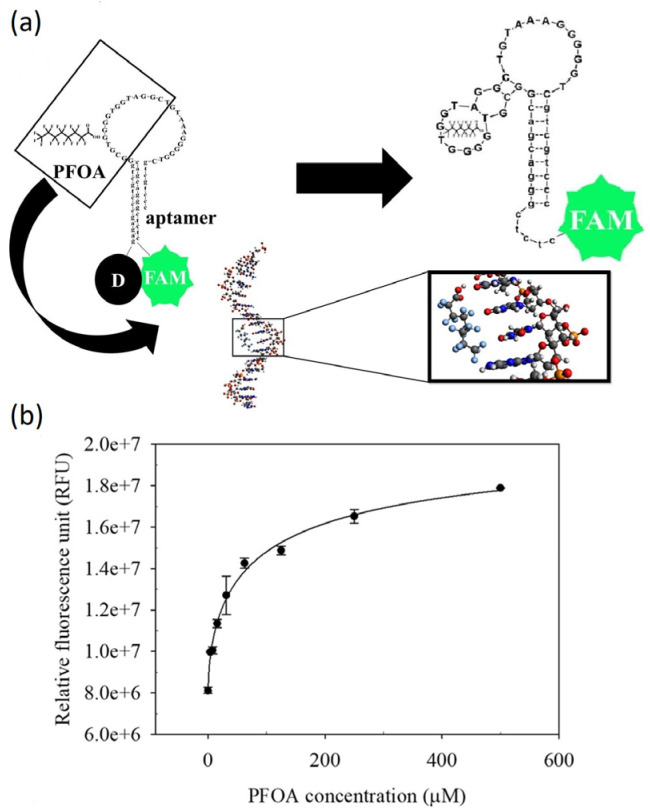
(**a**) The predicted 2D structures of aptamer after exposure to PFOA. (**b**) Fluorescence responses for binding of PFOA with different concentrations to the aptamer. The aptamer was modified with fluorescein (FAM) at 5′-end and dabcyl (D) at 3′-end, which was used as the quencher strand. Reproduced with permission from [60]. Copyright 2021, Elsevier (CC-BY-NC-ND).

Park et al. demonstrated the potential use of DNA aptamers for detecting PFAS molecules and other fluorinated alternatives for the first time in a study published in 2022 [59]. The designed aptamer was capable of specifically binding PFOA and was integrated into a fluorescence-based aptasensor, able to detect PFOA with a LoD of 0.17 μM in water. The detection mechanism was based on quenching of the fluorescence of fluorescein by dabcyl and, by binding of PFOA, the aptamer changed its conformation so that the quenching stopped. Figure 10a shows the predicted structure of the aptamer (with 30 bases) after binding to PFOA. The aptamer was mixed with PFOA solutions with different concentrations (0.5–50 μM) and, after 40 min, the fluorescence intensity was recorded. The fluorescence intensity was increasing with the increase in PFOA concentration (Figure 10b). The existence of interferents negligibly affected the aptamer performance, and the first proof of application was provided by testing the sensor in wastewater effluents. The fluorescence-based aptasensor was sufficiently sensitive for screening PFOA levels in water near accidental spills and industrial sites, where high concentrations of PFAS were anticipated. This work demonstrated the potential application of aptasensors for effective monitoring of the trace levels of different PFAS molecules and other fluorinated substances in water environments. The LoD is not yet low enough to measure concentrations below the regulatory limit (0.1 µg/L for each PFAS molecule and 0.24 nM for PFOA), but fluorescence-based sensors have the advantage that it is not necessary to immobilize the receptor on a solid support, everything can be performed in solution. To date, and to the best of our knowledge, there is no additional literature on PFAS-sensitive aptamers.

### 3.2. PFAS Sensing with Metal–Organic Frameworks

**Figure 11:**
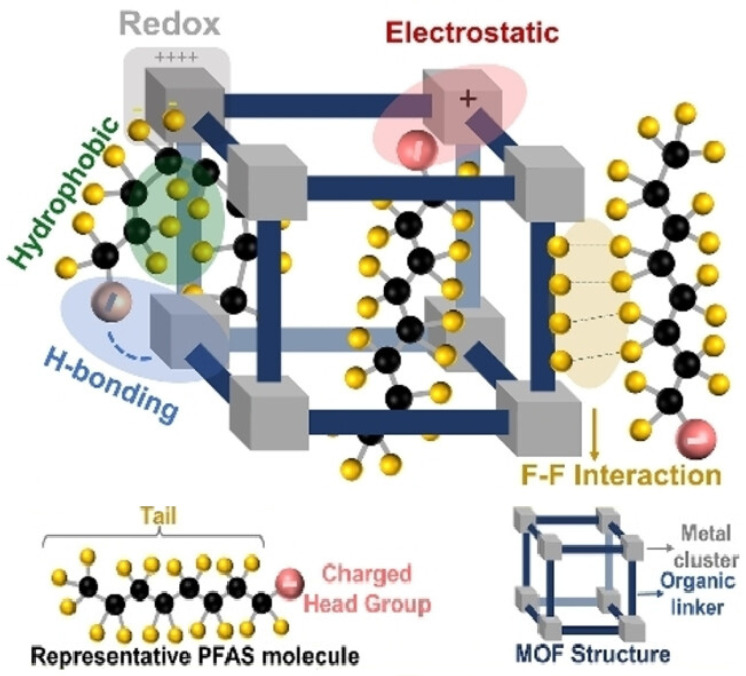
Schematic illustration of the different interactions between fluorinated MOFs and PFAS molecules including redox, electrostatic, hydrophobic, hydrogen bonding and F–F interactions. Reproduced with permission [66]. Copyright 2022, John Wiley and Sons (CC-BY-NC).

Metal–organic frameworks (MOFs) are a type of crystalline porous nanomaterials made of metal ions and organic ligands. Because of their large specific areas, tunable pore size, straightforward synthesis routes, abundant functional groups, and chemical stability, MOFs are extensively used in diverse fields including separation, gas storage, drug delivery, electrochemical applications, catalysis, and importantly the detection of chemicals [67]. They have been applied in affinity-based determination of various analytes such as alcohols, ammonia, biomolecules, and recently fluorocarbon [133,134,135]. Firstly, the enormous surface area (ranging from 10^3^ to 10^4^ m^2^ per gram of MOFs material) and porous structure of MOFs provide more interfaces and active sites for interaction with target molecules. Different types of interactions between MOFs and PFAS species exist, including redox, electrostatic, H-bonding, hydrophobic, and attractive intermolecular fluorine–fluorine (F–F) interactions as shown in Figure 11. Which interactions are at work depends on the precise type of the PFAS molecule and the design of the MOF structure. It is noteworthy that the negatively charged fluorine functionalities in two different molecules are responsible for an attractive force, which is known from experiments and quantum-chemistry calculations; see [Varadwaj, ChemPhysChem 2018]. Secondly, the organic ligands with versatile functional groups provide easy functionalization of MOFs with a broad range of molecules such as nucleic acids, enzymes, and nanoparticles. Finally, the diverse compositions of MOFs between metal and organic ligands offer a lot of functionality, such as catalytic activity, electrochemical activity, and optical activity. As a result, MOFs can be utilized as signal probes for different detection methods [136].

In 2020, Cheng and coworkers prepared a MOFs-based impedimetric sensor using a microfluidic platform for ultrasensitive in situ determination of PFOS [23]. The mesoporous MOFs Cr-MIL-101 (a chromium-based metal–organic framework) with high surface area and pore volumes was employed as the probe for capturing PFOS, which was based on the affinity of the chromium center toward both the fluorine and sulfonate functionalities. The MOFs capture probes were sandwiched between interdigitated microelectrodes in a microfluidic channel, forming an impedance sensor in a portable microfluidic device. This sensor directly measured PFOS concentrations by a proportional change in the electrical current as seen from the increase in the impedance signal. This microfluidic platform integrated with a MOFs-based sensor demonstrated ultra-sensitivity for the rapid in situ detection of PFOS with a LoD of 0.5 ng/L, corresponding to 1 pM at the molar scale. However, the selectivity of sensor towards other PFAS molecules was not yet studied.

**Figure 12:**
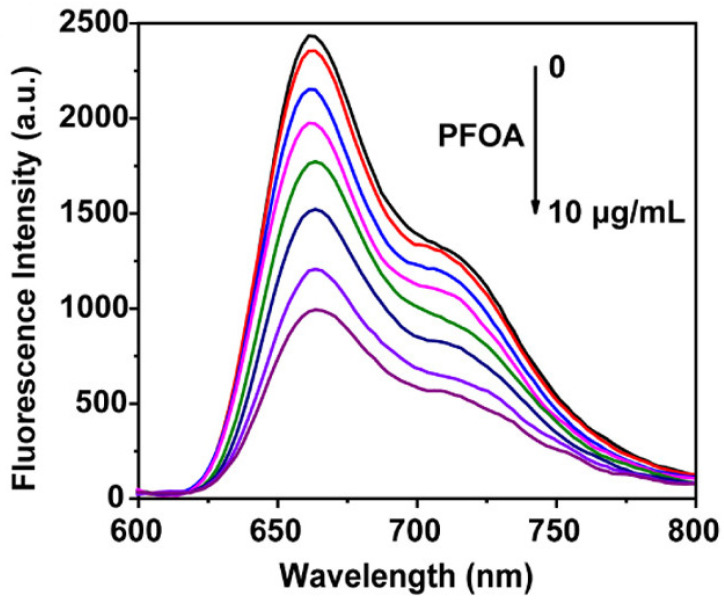
Fluorescence emission of the PCNs suspension at excitation wavelength λ_ex_ = 430 nm upon exposure to different concentrations of PFOA in water (0–10 μg/mL). Figure adopted from [137]. Copyright 2021, with permission from American Chemical Society.

Chen et al. designed a fluorescent MOFs array for optical sensing of multiple PFAS molecules in water samples [137]. The sensor array comprised three zirconium based porphyrinic coordination networks (PCNs) to determine PFAS molecules. The MOFs sensing array was also utilized to discriminate between six different PFAS by making a distinctive fluorescent response pattern for each molecule, according to their adsorptive affinity with the MOFs. The principal sensing mechanism was the quenching of the fluorescence emission of PCNs caused by the adsorption of PFAS. As an example, with increase in the PFOA concentration, the fluorescence emission of PCNs was quenched proportionally; see Figure 12. The calculated LoD for PFOA was 111 nM and it was in the same range for other PFAS molecules including PFOS. Importantly, the PCNs sensors showed a very fast response toward PFAS within only 10 s, owing to the ordered pore structure enabling rapid PFAS diffusion.

Several other PFAS sensors based on MOFs exist, which are summarized in Table 2; a few of these platforms are able to detect PFAS in complex samples in the relevant concentration ranges. Despite these promising results, there are still some challenges associated with the use of MOFs in the sensing of PFAS species. The synthesis process generally requires harsh solvents and high temperatures, and thus, a “greener” synthesis approach should be applied. Furthermore, most MOFs are not stable in aqueous media, which will limit their applicability in sensing platforms. If these challenges can be overcome, MOFs may prove to be exceptionally advantageous towards solving the difficulties associated with PFAS pollution.

## 4. Comparison between the Different Receptor Types

In the previous sections, a summary of recent studies on PFAS detection was provided. Different targets, receptor material, receptor type, readout principle, limit of detection, and sample type were discussed. MIP-based sensors seem to have the lowest LoDs when comparing them to other biomimetic PFAS sensing platforms. However, several challenges still lie ahead when it comes to incorporating these receptors into commercial devices. Real-world samples such as lake and river water contain very low concentrations of PFAS. Most of the user-friendly, handheld sensors can simply not reach the desired detection limits yet. Some of the more sensitive sensors on the other hand, are mostly focusing on the detection of PFOS and PFOA specifically, while there are more than 5000 different PFAS compounds identified [142]. Therefore, it is crucial to try to re-engineer these sensors towards the detection of a broader range of PFAS depending on the application. The next research phase should experiment with selectivity and intelligently design MIPs based on the envisioned application by integrating, e.g., computational studies into the design cycle [143].

The main challenge for industrialization, however, lays in upscaling the synthesis procedure towards mass production. In this regard, MIP-based receptors are more suitable for upscaling to industrial production due to their relatively low-cost and straightforward synthesis process. For all the receptors discussed in this review, it is essential to create large batches of sensors that are re-usable and provide accurate results in a reproducible manner. In a final step, the current lab-based prototypes should then be turned into handheld sensor solutions for on-site screening, combining for instance a dipstick-like sampler with a portable, smartphone-based transducer. The detection limits of the resulting sensors can be further optimized by combining biomimetic receptors with the most recent advances in the field of electrochemical and optical (quantum dots, fluorescence, etc.) MIP-based sensing [144,145,146].

## 5. Conclusions

PFAS molecules have attracted considerable attention worldwide as emerging pollutants because of their adverse effect on humans, aquatic life, and the environment. This review was written to provide readers with an overview of PFAS sensors based on synthetic receptors as these have the same benefits but overcome some of the drawbacks of natural bioreceptors. It summarizes different receptor layers used for selective determination of PFAS in the past few years.

MIP-based receptors are promising candidates, owing to their distinct ability to bind special targets with high selectivity. The sensors employing these receptors offer advantages with regard to their facile preparation, portability, user-friendliness, and cost-effectiveness. Synthesis of MIP-based receptors is possible via different approaches ranging from conventional polymerization methods such as bulk polymerization, to more advanced methods like electropolymerization. In many studies, nanomaterials have been used as substrate for electropolymerization improve the detection performance and response time of the sensor. In general, the MIP-based sensors demonstrate adequate sensing performance in terms of very low detection limit. Furthermore, many of the MIP-based sensing platforms introduced in this review show promising results for PFAS determination in real-world samples such as river water and tap water.

On the other hand, aptamers can also be used as recognition elements for PFAS detection but their detection limit needs to be lowered. There is only one publication on aptamers so far, dating from 2022, but more results in this context maybe expected in near future. Finally, the diverse compositions of MOFs between metal and organic ligands offer a lot of functionalities, making them promising candidates as signal probes for PFAS detection. Although using MOFs for PFAS determination is new, several recent studies show their capability at achieving highly sensitive and selective sensors for different PFAS species.

Considering all elements, both MIPs and MOFs are promising candidates to serve as receptors in on-site biomimetic PFAS sensors: both receptor types enable quantifying even subnanomolar concentrations, i.e., below the legally allowed limits. We see a slight advantage for MIPs because there are synthesis routes that enable continuous operation (nanoMIPs), or to fabricate large batches simultaneously and directly on transducer elements (electropolymerization). Moreover, it is facile to imprint MIP materials with a mixture of different PFAS molecules, so that the sensor will respond to a broad spectrum of these compounds. In the case of a positive sensor response, it will still be possible to perform a more selective analysis by chromatography and mass spectrometry. Such reduced selectivity can probably also be achieved by MOFs thanks to the fluorine–fluorine interaction. It is noteworthy that MOFs and MIPs can already be purchased from commercial suppliers and the step is small to adapt these materials towards PFAS detection. For on-site analysis with the analytical result ready within a few minutes, it is of course mandatory to make compact and low cost, but still accurate, readout techniques available. Here, we see a role for miniature photospectrometers and impedance analyzers. The costs have dropped tremendously in recent years and both transducers can be readout with a smartphone to arrive at a truly mobile application. A final element to bring sensor-based PFAS detection and quantification to the market would be accreditation of the instrument according to the norms of the International Organization for Standardization ISO. This still appears as a hurdle, but if sensor developers can show that sensor-derived data comply with the results of ISO-certified methods, this should become feasible. 

## Figures and Tables

**Table 1:** Comparison of the different MIP-based receptors for PFAS detection.

Target	Receptor Material	Receptor Type	Readout Principle	Limit of Detection	Sample Type	Ref.
PFOA	poly acrylamide	MIPs	HTM	22 pM	river water and soil extract	[104]
	poly VBT and PFDA		SPR sensor	2 pM	seawater	[11]
	CdTe@CdS/poly APTES		photoluminescence sensor	25 nM	river water and tap water	[127]
	AgI–BiOINFs/poly acrylamide		photoelectrochemical sensor	24 pM	river water and tap water	[8]
	poly pyrrole/ graphitic carbon nitride nanosheets		Electrochemi- luminescence sensor	24 pM	river water, tap water, and lake water	[41]
	poly pyrrole/Co/Fe@CNF	MIPs and MOFs	DPV	1.07 nM	wastewater	[67]
HFPO-DA	poly o-PD/gold electrode	MIPs	DPV	250 fM	river water	[92]
PFOS	poly APTES/SiO_2_ NPs nanoparticles	MIPs	fluorescence quantification	11 nM	river water and tap water	[119]
	TiO_2_ nanotube arrays/poly APTES		photoelectrochemical sensor	172 nM	river water, tap water, and mountain water	[128]
	polyaniline on paper		DC resistance measurements	2.4 pM	DI water	[46]
	phenolic resin		LC–MS/MS	12 pM	milk	[97]
	poly o-PD/GCE		DPV	0.05 nM	DI water	[2]
	G-UCNPs-SiO_2_/poly APTES		fluorescence quantification	1 pM	human serum, egg, lake water	[89]
	Au/poly o-PD		DPV	0.04 nM	tap water	[94]
	poly o-PD/ AuNS/GCE		DPV	0.015 nM	tap water	[125]
	poly o-PD/GCE		EIS	3.4 pM	river water	[93]
	poly DA/AuNPs/GCE		DPV	4.2 nM	lake water, canal water, tap water	[126]
	CNW/poly o-PD		DPV and EIS	2.4 nM	tap and wastewater	[91]
	poly chitosan/carbon quantum dots		fluorescence spectrophotometry	0.8 fM	serum and urine	[31]

Abbreviations: HTM: heat transfer method; VBT: (Vinylbenzyl) trimethylammonium chloride; PFDA: 1H,1H,2H,2H-perfluorodecyl acrylate; SPR: surface plasmon resonance; APTES: 3-aminopropyltriethoxysilane; AgI–BiOINFs: AgI nanoparticles–BiOI nanoflake arrays; DC: direct current; DPV: differential pulse voltammetry; HFPO-DA: hexafluoropropylene oxide dimer acid; EIS: electrochemical impedance spectroscopy; CNW: B,N-codoped carbon nanowalls; LC–MS/MS: liquid chromatography–tandem mass spectrometry; Co-N-C: cobalt-embedded Nitrogen-doped Carbon.

**Table 2:** Comparison of the different receptors based on MOFs and aptamer for PFAS detection.

Target	Receptor Material	Receptor Type	Readout Principle	Limit of Detection	Sample Type	Ref.
PFOA	DNA aptamer	aptamer	fluorescent quantification	0.17 μM	wastewater	[60]
	MOFs-coated probes	MOFs	mass spectrometry	26 pM	tap water, rainwater, and seawater	[138]
PFOS	MOFs Cr-MIL-10	MOFs	EIS	1 pM	groundwater	[23]
	zinc based MOFs		mass spectroscopy	1.28 nM	tap water and river water	[139]
	MOFs-derived Co-N-C nanosheets		colorimetric measurements	20 nM	river water, tap water, and lake water	[140]
PFAS	MIL-101(Cr)	MOFs	UHPLC–MS/MS	0.004– 0.12 ng/L	tap water, river water, wastewater	[141]
	zirconium based porphyrinic coordination networks		fluorescent quantification	111 nM for PFOA	surface water and groundwater	[137]

Abbreviations: UHPLC–MS/MS: ultra-high-performance liquid chromatography–tandem mass spectrometry; Co-N-C: cobalt-embedded nitrogen-doped carbon.

## References

[1] Chen C., Wang J., Yang S., Yan Z., Cai Q., Yao S. (2013). Analysis of Perfluorooctane Sulfonate and Perfluorooctanoic Acid with a Mixed-Mode Coating-Based Solid-Phase Microextraction Fiber. Talanta.

[2] Kazemi R., Potts E.I., Dick J.E. (2020). Quantifying Interferent Effects on Molecularly Imprinted Polymer Sensors for Per- and Polyfluoroalkyl Substances (PFAS). Anal. Chem..

[3] Smaili H., Ng C. (2022). Adsorption as a Remediation Technology for Short-Chain per- and Polyfluoroalkyl Substances (PFAS) from Water—A Critical Review. Environ. Sci..

[4] Yu H., Chen Y.F., Guo H.Q., Ma W.T., Li J., Zhou S.G., Lin S., Yan L.S., Li K.X. (2019). Preparation of Molecularly Imprinted Carbon Microspheres by One-Pot Hydrothermal Method and Their Adsorption Properties to Perfluorooctane Sulfonate. Chin. J. Anal. Chem..

[5] Hassan M.H., Khan R., Andreescu S. (2022). Advances in Electrochemical Detection Methods for Measuring Contaminants of Emerging Concerns. Electrochem. Sci. Adv..

[6] Jones J.L., Burket S.R., Hanley A., Shoemaker J.A. (2022). Development of a Standardized Adsorbable Organofluorine Screening Method for Wastewaters with Detection by Combustion Ion Chromatography. Anal. Methods.

[7] Bell E.M., De Guise S., McCutcheon J.R., Lei Y., Levin M., Li B., Rusling J.F., Lawrence D.A., Cavallari J.M., O’Connell C. (2021). Exposure, Health Effects, Sensing, and Remediation of the Emerging PFAS Contaminants—Scientific Challenges and Potential Research Directions. Sci. Total Environ..

[8] Gong J., Fang T., Peng D., Li A., Zhang L. (2015). A Highly Sensitive Photoelectrochemical Detection of Perfluorooctanic Acid with Molecularly Imprined Polymer-Functionalized Nanoarchitectured Hybrid of AgI-BiOI Composite. Biosens. Bioelectron..

[9] Ranaweera R., An S., Cao Y., Luo L. (2022). Highly Efficient Preconcentration Using Anodically Generated Shrinking Gas Bubbles for Per- and Polyfluoroalkyl Substances (PFAS) Detection. Anal. Bioanal. Chem..

[10] Cao F., Wang L., Ren X., Wu F., Sun H., Lu S. (2018). The Application of Molecularly Imprinted Polymers in Passive Sampling for Selective Sampling Perfluorooctanesulfonic Acid and Perfluorooctanoic Acid in Water Environment. Environ. Sci. Pollut. Res..

[11] Pitruzzella R., Arcadio F., Perri C., Del Prete D., Porto G., Zeni L., Cennamo N. (2023). Ultra-Low Detection of Perfluorooctanoic Acid Using a Novel Plasmonic Sensing Approach Combined with Molecularly Imprinted Polymers. Chemosensors.

[12] Hill N.I., Becanova J., Lohmann R. (2022). A Sensitive Method for the Detection of Legacy and Emerging Per- and Polyfluorinated Alkyl Substances (PFAS) in Dairy Milk. Anal. Bioanal. Chem..

[13] Directive (EU) 2020/2184 of the European Parliament and of the Council of 16 December 2020 on the Quality of Water Intended for Human Consumption (Recast). https://Eur-Lex.Europa.Eu/Eli/Dir/2020/2184/Oj.

[14] Fenton S.E., Ducatman A., Boobis A., DeWitt J.C., Lau C., Ng C., Smith J.S., Roberts S.M. (2021). Per- and Polyfluoroalkyl Substance Toxicity and Human Health Review: Current State of Knowledge and Strategies for Informing Future Research. Environ. Toxicol. Chem..

[15] Skaggs C.S., Logue B.A. (2021). Ultratrace Analysis of Per- and Polyfluoroalkyl Substances in Drinking Water Using Ice Concentration Linked with Extractive Stirrer and High Performance Liquid Chromatography—Tandem Mass Spectrometry. J. Chromatogr. A.

[16] Casey J.S., Jackson S.R., Ryan J., Newton S.R. (2023). The Use of Gas Chromatography—High Resolution Mass Spectrometry for Suspect Screening and Non-Targeted Analysis of per- and Polyfluoroalkyl Substances. J. Chromatogr. A.

[17] Gogoi P., Yao Y., Li Y.C. (2023). Understanding PFOS Adsorption on a Pt Electrode for Electrochemical Sensing Applications. ChemElectroChem.

[18] Gonzalez de Vega R., Cameron A., Clases D., Dodgen T.M., Doble P.A., Bishop D.P. (2021). Simultaneous Targeted and Non-Targeted Analysis of per- and Polyfluoroalkyl Substances in Environmental Samples by Liquid Chromatography-Ion Mobility-Quadrupole Time of Flight-Mass Spectrometry and Mass Defect Analysis. J. Chromatogr. A.

[19] Farooq S., Nie J., Cheng Y., Yan Z., Li J., Bacha S.A.S., Mushtaq A., Zhang H. (2018). Molecularly Imprinted Polymers’ Application in Pesticide Residue Detection. Analyst.

[20] Tarannum N., Khatoon S., Dzantiev B.B. (2020). Perspective and Application of Molecular Imprinting Approach for Antibiotic Detection in Food and Environmental Samples: A Critical Review. Food Control.

[21] Oprea A., Weimar U. (2019). Gas Sensors Based on Mass-Sensitive Transducers Part 1: Transducers and Receptors—Basic Understanding. Anal. Bioanal. Chem..

[22] Lian X., Zhou Y.J., Zhang H.F., Li M., Huang X.C. (2020). Luminescence Turn-on Detection by an Entanglement-Protected MOF Operating: Via a Divided Receptor-Transducer Protocol. J. Mater. Chem. C.

[23] Cheng Y.H., Barpaga D., Soltis J.A., Shutthanandan V., Kargupta R., Han K.S., McGrail B.P., Motkuri R.K., Basuray S., Chatterjee S. (2020). Metal-Organic Framework-Based Microfluidic Impedance Sensor Platform for Ultrasensitive Detection of Perfluorooctanesulfonate. ACS Appl. Mater. Interfaces.

[24] Naseri M., Mohammadniaei M., Sun Y., Ashley J. (2020). The Use of Aptamers and Molecularly Imprinted Polymers in Biosensors for Environmental Monitoring: A Tale of Two Receptors. Chemosensors.

[25] Berhanu A., Mutanda I., Taolin J., Qaria M.A., Yang B., Zhu D. (2023). A Review of Microbial Degradation of Per- and Polyfluoroalkyl Substances (PFAS): Biotransformation Routes and Enzymes. Sci. Total Environ..

[26] Shahsavari E., Rouch D., Khudur L.S., Thomas D., Aburto-Medina A., Ball A.S. (2021). Challenges and Current Status of the Biological Treatment of PFAS-Contaminated Soils. Front. Bioeng. Biotechnol..

[27] Ali G.K., Omer K.M. (2022). Molecular Imprinted Polymer Combined with Aptamer (MIP-Aptamer) as a Hybrid Dual Recognition Element for Bio(Chemical) Sensing Applications. Review. Talanta.

[28] Kamyab H., Chelliapan S., Tavakkoli O., Mesbah M., Bhutto J.K., Khademi T., Kirpichnikova I., Ahmad A., ALJohani A.A. (2022). A Review on Carbon-Based Molecularly-Imprinted Polymers (CBMIP) for Detection of Hazardous Pollutants in Aqueous Solutions. Chemosphere.

[29] Ashley J., Shahbazi M.A., Kant K., Chidambara V.A., Wolff A., Bang D.D., Sun Y. (2017). Molecularly Imprinted Polymers for Sample Preparation and Biosensing in Food Analysis: Progress and Perspectives. Biosens. Bioelectron..

[30] Rebelo P., Costa-Rama E., Seguro I., Pacheco J.G., Nouws H.P.A., Cordeiro M.N.D.S., Delerue-Matos C. (2021). Molecularly Imprinted Polymer-Based Electrochemical Sensors for Environmental Analysis. Biosens. Bioelectron..

[31] Jiao Z., Li J., Mo L., Liang J., Fan H. (2018). A Molecularly Imprinted Chitosan Doped with Carbon Quantum Dots for Fluorometric Determination of Perfluorooctane Sulfonate. Microchim. Acta.

[32] Yu Q., Deng S., Yu G. (2008). Selective Removal of Perfluorooctane Sulfonate from Aqueous Solution Using Chitosan-Based Molecularly Imprinted Polymer Adsorbents. Water Res..

[33] Malik A.A., Nantasenamat C., Piacham T. (2017). Molecularly Imprinted Polymer for Human Viral Pathogen Detection. Mater. Sci. Eng. C.

[34] Cennamo N., D’Agostino G., Sequeira F., Mattiello F., Porto G., Biasiolo A., Nogueira R., Bilro L., Zeni L. (2018). A Simple and Low-Cost Optical Fiber Intensity-Based Configuration for Perfluorinated Compounds in Water Solution. Sensors.

[35] Selvolini G., Marrazza G. (2017). MIP-Based Sensors: Promising New Tools for Cancer Biomarker Determination. Sensors.

[36] Peeters M., Troost F.J., van Grinsven B., Horemans F., Alenus J., Murib M.S., Keszthelyi D., Ethirajan A., Thoelen R., Cleij T.J. (2012). MIP-Based Biomimetic Sensor for the Electronic Detection of Serotonin in Human Blood Plasma. Sens. Actuators B Chem..

[37] Cao Y., Feng T., Xu J., Xue C. (2019). Recent Advances of Molecularly Imprinted Polymer-Based Sensors in the Detection of Food Safety Hazard Factors. Biosens. Bioelectron..

[38] Whitcombe M.J., Chianella I., Larcombe L., Piletsky S.A., Noble J., Porter R., Horgan A. (2011). The Rational Development of Molecularly Imprinted Polymer-Based Sensors for Protein Detection. Chem. Soc. Rev..

[39] Cao F., Wang L., Ren X., Sun H. (2016). Synthesis of a Perfluorooctanoic Acid Molecularly Imprinted Polymer for the Selective Removal of Perfluorooctanoic Acid in an Aqueous Environment. J. Appl. Polym. Sci..

[40] Fang C., Chen Z., Megharaj M., Naidu R. (2016). Potentiometric Detection of AFFFs Based on MIP. Environ. Technol. Innov..

[41] Chen S., Li A., Zhang L., Gong J. (2015). Molecularly Imprinted Ultrathin Graphitic Carbon Nitride Nanosheets-Based Electrochemiluminescence Sensing Probe for Sensitive Detection of Perfluorooctanoic Acid. Anal. Chim. Acta.

[42] Dickman R.A., Aga D.S. (2022). A Review of Recent Studies on Toxicity, Sequestration, and Degradation of per- and Polyfluoroalkyl Substances (PFAS). J. Hazard. Mater..

[43] Tasfaout A., Ibrahim F., Morrin A., Brisset H., Sorrentino I., Nanteuil C., Laffite G., Nicholls I.A., Regan F., Branger C. (2023). Molecularly Imprinted Polymers for Per- and Polyfluoroalkyl Substances Enrichment and Detection. Talanta.

[44] Ganesan S., Chawengkijwanich C., Gopalakrishnan M., Janjaroen D. (2022). Detection Methods for Sub-Nanogram Level of Emerging Pollutants—Per and Polyfluoroalkyl Substances. Food Chem. Toxicol..

[45] Pardeshi S., Dhodapkar R. (2022). Advances in Fabrication of Molecularly Imprinted Electrochemical Sensors for Detection of Contaminants and Toxicants. Environ. Res..

[46] Chi T.Y., Chen Z., Kameoka J. (2020). Perfluorooctanesulfonic Acid Detection Using Molecularly Imprinted Polyaniline on a Paper Substrate. Sensors.

[47] Vu O.T., Nguyen Q.H., Nguy Phan T., Luong T.T., Eersels K., Wagner P., Truong L.T.N. (2023). Highly Sensitive Molecularly Imprinted Polymer-Based Electrochemical Sensors Enhanced by Gold Nanoparticles for Norfloxacin Detection in Aquaculture Water. ACS Omega.

[48] Lowdon J.W., Eersels K., Arreguin-Campos R., Caldara M., Heidt B., Rogosic R., Jimenez-Monroy K.L., Cleij T.J., Diliën H., van Grinsven B. (2020). A Molecularly Imprinted Polymer-Based Dye Displacement Assay for the Rapid Visual Detection of Amphetamine in Urine. Molecules.

[49] Cennamo N., D’Agostino G., Porto G., Biasiolo A., Perri C., Arcadio F., Zeni L. (2018). A Molecularly Imprinted Polymer on a Plasmonic Plastic Optical Fiber to Detect Perfluorinated Compounds in Water. Sensors.

[50] Ayerdurai V., Cieplak M., Kutner W. (2023). Molecularly Imprinted Polymer-Based Electrochemical Sensors for Food Contaminants Determination. Trends Anal. Chem..

[51] Jahanban-Esfahlan A., Roufegarinejad L., Jahanban-Esfahlan R., Tabibiazar M., Amarowicz R. (2020). Latest Developments in the Detection and Separation of Bovine Serum Albumin Using Molecularly Imprinted Polymers. Talanta.

[52] Jamalipour Soufi G., Iravani S., Varma R.S. (2021). Molecularly Imprinted Polymers for the Detection of Viruses: Challenges and Opportunities. Analyst.

[53] McClements J., Bar L., Singla P., Canfarotta F., Thomson A., Czulak J., Johnson R.E., Crapnell R.D., Banks C.E., Payne B. (2022). Molecularly Imprinted Polymer Nanoparticles Enable Rapid, Reliable, and Robust Point-of-Care Thermal Detection of SARS-CoV-2. ACS Sens..

[54] Stilman W., Campolim Lenzi M., Wackers G., Deschaume O., Yongabi D., Mathijssen G., Bartic C., Gruber J., Wübbenhorst M., Heyndrickx M. (2022). Low Cost, Sensitive Impedance Detection of *E. coli* Bacteria in Food-Matrix Samples Using Surface-Imprinted Polymers as Whole-Cell Receptors. Phys. Status Solidi A.

[55] Eersels K., Lieberzeit P., Wagner P. (2016). A Review on Synthetic Receptors for Bioparticle Detection Created by Surface-Imprinting Techniques—From Principles to Applications. ACS Sens..

[56] Seo H.B., Gu M.B. (2017). Aptamer-Based Sandwich-Type Biosensors. J. Biol. Eng..

[57] Zhou W., Jimmy Huang P.J., Ding J., Liu J. (2014). Aptamer-Based Biosensors for Biomedical Diagnostics. Analyst.

[58] Kudłak B., Wieczerzak M. (2020). Aptamer Based Tools for Environmental and Therapeutic Monitoring: A Review of Developments, Applications, Future Perspectives. Crit. Rev. Environ. Sci. Technol..

[59] Park J., Yang K.A., Choi Y., Choe J.K. (2022). Novel ssDNA Aptamer-Based Fluorescence Sensor for Perfluorooctanoic Acid Detection in Water. Environ. Int..

[60] Mukunzi D., Habimana J.d.D., Li Z., Zou X. (2022). Mycotoxins Detection: View in the Lens of Molecularly Imprinted Polymer and Nanoparticles. Crit. Rev. Food Sci. Nutr..

[61] Vanoursouw T.M., Rottiger T., Wadzinski K.A., Vanderwaal B.E., Snyder M.J., Bittner R.T., Farha O.K., Riha S.C., Mondloch J.E. (2023). Adsorption of a PFAS Utilizing MOF-808: Development of an Undergraduate Laboratory Experiment in a Capstone Course. J. Chem. Educ..

[62] Li R., Alomari S., Stanton R., Wasson M.C., Islamoglu T., Farha O.K., Holsen T.M., Thagard S.M., Trivedi D.J., Wriedt M. (2021). Efficient Removal of Per- And Polyfluoroalkyl Substances from Water with Zirconium-Based Metal-Organic Frameworks. Chem. Mater..

[63] FitzGerald L.I., Olorunyomi J.F., Singh R., Doherty C.M. (2022). Towards Solving the PFAS Problem: The Potential Role of Metal-Organic Frameworks. ChemSusChem.

[64] Hu M.L., Razavi S.A.A., Piroozzadeh M., Morsali A. (2020). Sensing Organic Analytes by Metal-Organic Frameworks: A New Way of Considering the Topic. Inorg. Chem. Front..

[65] Menger R.F., Funk E., Henry C.S., Borch T. (2021). Sensors for Detecting Per- and Polyfluoroalkyl Substances (PFAS): A Critical Review of Development Challenges, Current Sensors, and Commercialization Obstacles. Chem. Eng. J..

[66] Karbassiyazdi E., Kasula M., Modak S., Pala J., Kalantari M., Altaee A., Esfahani M.R., Razmjou A. (2023). A Juxtaposed Review on Adsorptive Removal of PFAS by Metal-Organic Frameworks (MOFs) with Carbon-Based Materials, Ion Exchange Resins, and Polymer Adsorbents. Chemosphere.

[67] Wang Y., Ren R., Chen F., Jing L., Tian Z., Li Z., Wang J., Hou C. (2023). Molecularly Imprinted MOFs-Driven Carbon Nanofiber for Sensitive Electrochemical Detection and Targeted Electro-Fenton Degradation of Perfluorooctanoic Acid. Sep. Purif. Technol..

[68] Pirot S.M., Omer K.M., Alshatteri A.H., Ali G.K., Shatery O.B.A. (2023). Dual-Template Molecularly Surface Imprinted Polymer on Fluorescent Metal-Organic Frameworks Functionalized with Carbon Dots for Ascorbic Acid and Uric Acid Detection. Spectrochim. Acta Part A Mol. Biomol. Spectrosc..

[69] Lv M., Zhou W., Tavakoli H., Bautista C., Xia J., Wang Z., Li X.J. (2021). Aptamer-Functionalized Metal-Organic Frameworks (MOFs) for Biosensing. Biosens. Bioelectron..

[70] Zhou Q., Xu Z., Liu Z. (2022). Molecularly Imprinting-Aptamer Techniques and Their Applications in Molecular Recognition. Biosensors.

[71] Wu Y., Li Y., Tian A., Mao K., Liu J. (2016). Selective Removal of Perfluorooctanoic Acid Using Molecularly Imprinted Polymer-Modified TiO_2_ Nanotube Arrays. Int. J. Photoenergy.

[72] Abbasian Chaleshtari Z., Foudazi R. (2022). A Review on Per- and Polyfluoroalkyl Substances (PFAS) Remediation: Separation Mechanisms and Molecular Interactions. ACS ES T Water.

[73] Karadurmus L., Bilge S., Sınağ A., Ozkan S.A. (2022). Molecularly Imprinted Polymer (MIP)-Based Sensing for Detection of Explosives: Current Perspectives and Future Applications. Trends Anal. Chem..

[74] Caldara M., van Wissen G., Cleij T.J., Diliën H., van Grinsven B., Eersels K., Lowdon J.W. (2023). Deposition Methods for the Integration of Molecularly Imprinted Polymers (MIPs) in Sensor Applications. Adv. Sens. Res..

[75] Cennamo N., D’Agostino G., Arcadio F., Perri C., Porto G., Biasiolo A., Zeni L. (2020). Measurement of MIPs Responses Deposited on Two SPR-POF Sensors Realized by Different Photoresist Buffer Layers. IEEE Trans. Instrum. Meas..

[76] Hasseb A.A., Abdel Ghani N.d.T., Shehab O.R., El Nashar R.M. (2022). Application of Molecularly Imprinted Polymers for Electrochemical Detection of Some Important Biomedical Markers and Pathogens. Curr. Opin. Electrochem..

[77] Metwally M.G., Benhawy A.H., Khalifa R.M., El Nashar R.M., Trojanowicz M. (2021). Application of Molecularly Imprinted Polymers in the Analysis of Waters and Wastewaters. Molecules.

[78] Jamieson O., Mecozzi F., Crapnell R.D., Battell W., Hudson A., Novakovic K., Sachdeva A., Canfarotta F., Herdes C., Banks C.E. (2021). Approaches to the Rational Design of Molecularly Imprinted Polymers Developed for the Selective Extraction or Detection of Antibiotics in Environmental and Food Samples. Phys. Status Solidi A.

[79] Irshad M., Iqbal N., Mujahid A., Afzal A., Hussain T., Sharif A., Ahmad E., Athar M.M. (2013). Molecularly Imprinted Nanomaterials for Sensor Applications. Nanomaterials.

[80] Yu H., Chen H., Fang B., Sun H. (2023). Sorptive Removal of Per- and Polyfluoroalkyl Substances from Aqueous Solution: Enhanced Sorption, Challenges and Perspectives. Sci. Total Environ..

[81] Crapnell R.D., Hudson A., Foster C.W., Eersels K., van Grinsven B., Cleij T.J., Banks C.E., Peeters M. (2019). Recent Advances in Electrosynthesized Molecularly Imprinted Polymer Sensing Platforms for Bioanalyte Detection. Sensors.

[82] Wackers G., Cornelis P., Putzeys T., Peeters M., Tack J., Troost F., Doll T., Verhaert N., Wagner P. (2021). Electropolymerized Receptor Coatings for the Quantitative Detection of Histamine with a Catheter-Based, Diagnostic Sensor. ACS Sens..

[83] Yang J., Li Y., Wang J., Sun X., Cao R., Sun H., Huang C., Chen J. (2015). Molecularly Imprinted Polymer Microspheres Prepared by Pickering Emulsion Polymerization for Selective Solid-Phase Extraction of Eight Bisphenols from Human Urine Samples. Anal. Chim. Acta.

[84] Yang Y., Shen X. (2022). Preparation and Application of Molecularly Imprinted Polymers for Flavonoids: Review and Perspective. Molecules.

[85] Cui F., Zhou Z., Zhou H.S. (2020). Molecularly Imprinted Polymers and Surface Imprinted Polymers Based Electrochemical Biosensor for Infectious Diseases. Sensors.

[86] Caldara M., Lowdon J.W., Rogosic R., Arreguin-Campos R., Jimenez-Monroy K.L., Heidt B., Tschulik K., Cleij T.J., Diliën H., Eersels K. (2021). Thermal Detection of Glucose in Urine Using a Molecularly Imprinted Polymer as a Recognition Element. ACS Sens..

[87] Tretjakov A., Syritski V., Reut J., Boroznjak R., Volobujeva O., Öpik A. (2013). Surface Molecularly Imprinted Polydopamine Films for Recognition of Immunoglobulin G. Microchim. Acta.

[88] Stilman W., Yongabi D., Bakhshi Sichani S., Thesseling F., Deschaume O., Putzeys T., Pinto T.C., Verstrepen K., Bartic C., Wübbenhorst M. (2021). Detection of Yeast Strains by Combining Surface-Imprinted Polymers with Impedance-Based Readout. Sens. Actuators B Chem..

[89] Tian L., Guo H., Li J., Yan L., Zhu E., Liu X., Li K. (2021). Fabrication of a Near-Infrared Excitation Surface Molecular Imprinting Ratiometric Fluorescent Probe for Sensitive and Rapid Detecting Perfluorooctane Sulfonate in Complex Matrix. J. Hazard. Mater..

[90] Dong C., Shi H., Han Y., Yang Y., Wang R., Men J. (2021). Molecularly Imprinted Polymers by the Surface Imprinting Technique. Eur. Polym. J..

[91] Pierpaoli M., Szopińska M., Olejnik A., Ryl J., Fudala-Ksiażek S., Łuczkiewicz A., Bogdanowicz R. (2023). Engineering Boron and Nitrogen Codoped Carbon Nanoarchitectures to Tailor Molecularly Imprinted Polymers for PFOS Determination. J. Hazard. Mater..

[92] Glasscott M.W., Vannoy K.J., Kazemi R., Verber M.D., Dick J.E. (2020). μ-MIP: Molecularly Imprinted Polymer-Modified Microelectrodes for the Ultrasensitive Quantification of GenX (HFPO-DA) in River Water. Environ. Sci. Technol. Lett..

[93] Clark R.B., Dick J.E. (2020). Electrochemical Sensing of Perfluorooctanesulfonate (PFOS) Using Ambient Oxygen in River Water. ACS Sens..

[94] Karimian N., Stortini A.M., Moretto L.M., Costantino C., Bogialli S., Ugo P. (2018). Electrochemosensor for Trace Analysis of Perfluorooctanesulfonate in Water Based on a Molecularly Imprinted Poly(o-Phenylenediamine) Polymer. ACS Sens..

[95] Dery L., Zelikovich D., Mandler D. (2022). Electrochemistry of Molecular Imprinting of Large Entities. Curr. Opin. Electrochem..

[96] Mahmoudpour M., Torbati M., Mousavi M.M., de la Guardia M., Ezzati Nazhad Dolatabadi J. (2020). Nanomaterial-Based Molecularly Imprinted Polymers for Pesticides Detection: Recent Trends and Future Prospects. Trends Anal. Chem..

[97] Ren J., Lu Y., Han Y., Qiao F., Yan H. (2023). Novel Molecularly Imprinted Phenolic Resin–Dispersive Filter Extraction for Rapid Determination of Perfluorooctanoic Acid and Perfluorooctane Sulfonate in Milk. Food Chem..

[98] Mostafiz B., Bigdeli S.A., Banan K., Afsharara H., Hatamabadi D., Mousavi P., Hussain C.M., Keçili R., Ghorbani-Bidkorbeh F. (2021). Molecularly Imprinted Polymer-Carbon Paste Electrode (MIP-CPE)-Based Sensors for the Sensitive Detection of Organic and Inorganic Environmental Pollutants: A Review. Trends Environ. Anal. Chem..

[99] Peeters M., Troost F.J., Mingels R.H.G., Welsch T., van Grinsven B., Vranken T., Ingebrandt S., Thoelen R., Cleij T.J., Wagner P. (2013). Impedimetric Detection of Histamine in Bowel Fluids Using Synthetic Receptors with pH-Optimized Binding Characteristics. Anal. Chem..

[100] ul Gani Mir T., Malik A.Q., Singh J., Shukla S., Kumar D. (2022). An Overview of Molecularly Imprinted Polymers Embedded with Quantum Dots and Their Implementation as an Alternative Approach for Extraction and Detection of Crocin. ChemistrySelect.

[101] Akgönüllü S., Kılıç S., Esen C., Denizli A. (2023). Molecularly Imprinted Polymer-Based Sensors for Protein Detection. Polymers.

[102] Wang L., Zhi K., Zhang Y., Liu Y., Zhang L., Yasin A., Lin Q. (2019). Molecularly Imprinted Polymers for Gossypol via Sol-Gel, Bulk, and Surface Layer Imprinting-A Comparative Study. Polymers.

[103] Li T., Li X., Liu H., Deng Z., Zhang Y., Zhang Z., He Y., Yang Y., Zhong S. (2020). Preparation and Characterization of Molecularly Imprinted Polymers Based on β-Cyclodextrin-Stabilized Pickering Emulsion Polymerization for Selective Recognition of Erythromycin from River Water and Milk. J. Sep. Sci..

[104] Tabar F.A., Lowdon J.W., Caldara M., Cleij T.J., Wagner P., Diliën H., Eersels K., van Grinsven B. (2023). Thermal Determination of Perfluoroalkyl Substances in Environmental Samples Employing a Molecularly Imprinted Polyacrylamide as a Receptor Layer. Environ. Technol. Innov..

[105] van Grinsven B., Eersels K., Peeters M., Losada-Pérez P., Vandenryt T., Cleij T.J., Wagner P. (2014). The Heat-Transfer Method: A Versatile Low-Cost, Label-Free, Fast, and User-Friendly Readout Platform for Biosensor Applications. ACS Appl. Mater. Interfaces.

[106] Wagner P., Bakhshi Sichani S., Khorshid M., Lieberzeit P., Losada-Pérez P., Yongabi D. (2023). Bioanalytical Sensors Using the Heat-Transfer Method HTM and Related Techniques. tm-Tech. Mess..

[107] Lowdon J.W., Diliën H., van Grinsven B., Eersels K., Cleij T.J. (2021). Colorimetric Sensing of Amoxicillin Facilitated by Molecularly Imprinted Polymers. Polymers.

[108] Wang Z., Zhang Z., Yan R., Fu X., Wang G., Wang Y., Li Z., Zhang X., Hou J. (2021). Facile Fabrication of Snowman-like Magnetic Molecularly Imprinted Polymer Microspheres for Bisphenol A via One-Step Pickering Emulsion Polymerization. React. Funct. Polym..

[109] Chen H., Son S., Zhang F., Yan J., Li Y., Ding H., Ding L. (2015). Rapid Preparation of Molecularly Imprinted Polymers by Microwave-Assisted Emulsion Polymerization for the Extraction of Florfenicol in Milk. J. Chromatogr. B.

[110] Zhao G., Liu J., Liu M., Han X., Peng Y., Tian X., Liu J., Zhang S. (2020). Synthesis of Molecularly Imprinted Polymer via Emulsion Polymerization for Application in Solanesol Separation. Appl. Sci..

[111] Pardeshi S., Singh S.K. (2016). Precipitation Polymerization: A Versatile Tool for Preparing Molecularly Imprinted Polymer Beads for Chromatography Applications. RSC Adv..

[112] Alizadeh T., Memarbashi N. (2012). Evaluation of the Facilitated Transport Capabilities of Nano- and Micro-Sized Molecularly Imprinted Polymers (MIPs) in a Bulk Liquid Membrane System. Sep. Purif. Technol..

[113] Rehman A.U., Crimi M., Andreescu S. (2023). Current and Emerging Analytical Techniques for the Determination of PFAS in Environmental Samples. Trends Environ. Anal. Chem..

[114] Islam G.J., Arrigan D.W.M. (2022). Voltammetric Selectivity in Detection of Ionized Perfluoroalkyl Substances at Micro-Interfaces between Immiscible Electrolyte Solutions. ACS Sens..

[115] Clark R.B., Dick J.E. (2021). Towards Deployable Electrochemical Sensors for Per- And Polyfluoroalkyl Substances (PFAS). ChemComm.

[116] Moro G., Cristofori D., Bottari F., Cattaruzza E., De Wael K., Moretto L.M. (2019). Redesigning an Electrochemical MIP Sensor for PFOS: Practicalities and Pitfalls. Sensors.

[117] Chi H., Liu G. (2023). Carbon Nanomaterial-Based Molecularly Imprinted Polymer Sensors for Detection of Hazardous Substances in Food: Recent Progress and Future Trends. Food Chem..

[118] Gao M., Gao Y., Chen G., Huang X., Xu X., Lv J., Wang J., Xu D., Liu G. (2020). Recent Advances and Future Trends in the Detection of Contaminants by Molecularly Imprinted Polymers in Food Samples. Front. Chem..

[119] Feng H., Wang N., Trant T., Yuan L., Li J., Cai Q. (2014). Surface Molecular Imprinting on Dye-(NH_2_)-SiO_2_ NPs for Specific Recognition and Direct Fluorescent Quantification of Perfluorooctane Sulfonate. Sens. Actuators B Chem..

[120] Steigerwald J.M., Peng S., Ray J.R. (2022). Novel Perfluorooctanesulfonate-Imprinted Polymer Immobilized on Spent Coffee Grounds Biochar for Selective Removal of Perfluoroalkyl Acids in Synthetic Wastewater. ACS EST Eng..

[121] Du L., Wu Y., Zhang X., Zhang F., Chen X., Cheng Z., Wu F., Tan K. (2017). Preparation of Magnetic Molecularly Imprinted Polymers for the Rapid and Selective Separation and Enrichment of Perfluorooctane Sulfonate. J. Sep. Sci..

[122] Lin L., Guo H., Lin S., Chen Y., Yan L., Zhu E., Li K. (2021). Selective Extraction of Perfluorooctane Sulfonate in Real Samples by Superparamagnetic Nanospheres Coated with a Polydopamine-Based Molecularly Imprinted Polymer. J. Sep. Sci..

[123] Du L., Cheng Z., Zhu P., Chen Q., Wu Y., Tan K. (2018). Preparation of Mesoporous Silica Nanoparticles Molecularly Imprinted Polymer for Efficient Separation and Enrichment of Perfluorooctane Sulfonate. J. Sep. Sci..

[124] Guo H., Liu Y., Ma W., Yan L., Li K., Lin S. (2018). Surface Molecular Imprinting on Carbon Microspheres for Fast and Selective Adsorption of Perfluorooctane Sulfonate. J. Hazard. Mater..

[125] Lu D., Zhu D.Z., Gan H., Yao Z., Luo J., Yu S., Kurup P. (2022). An Ultra-Sensitive Molecularly Imprinted Polymer (MIP) and Gold Nanostars (AuNS) Modified Voltammetric Sensor for Facile Detection of Perfluorooctance Sulfonate (PFOS) in Drinking Water. Sens. Actuators B Chem..

[126] Gao Y., Gou W., Zeng W., Chen W., Jiang J., Lu J. (2023). Determination of Perfluorooctanesulfonic Acid in Water by Polydopamine Molecularly Imprinted/Gold Nanoparticles Sensor. Microchem. J..

[127] Zheng L., Zheng Y., Liu Y., Long S., Du L., Liang J., Huang C., Swihart M.T., Tan K. (2019). Core-Shell Quantum Dots Coated with Molecularly Imprinted Polymer for Selective Photoluminescence Sensing of Perfluorooctanoic Acid. Talanta.

[128] Tran T.T., Li J., Feng H., Cai J., Yuan L., Wang N., Cai Q. (2014). Molecularly Imprinted Polymer Modified TiO_2_ Nanotube Arrays for Photoelectrochemical Determination of Perfluorooctane Sulfonate (PFOS). Sens. Actuators B Chem..

[129] Yang S., Teng Y., Cao Q., Bai C., Fang Z., Xu W. (2019). Electrochemical Sensor Based on Molecularly Imprinted Polymer-Aptamer Hybrid Receptor for Voltammetric Detection of Thrombin. J. Electrochem. Soc..

[130] Hayat A., Marty J.L. (2014). Aptamer Based Electrochemical Sensors for Emerging Environmental Pollutants. Front. Chem..

[131] Karimzadeh Z., Mahmoudpour M., Guardia M.d.l., Ezzati Nazhad Dolatabadi J., Jouyban A. (2022). Aptamer-Functionalized Metal Organic Frameworks as an Emerging Nanoprobe in the Food Safety Field: Promising Development Opportunities and Translational Challenges. Trends Anal. Chem..

[132] Wolter O., Mayer G. (2017). Aptamers as Valuable Molecular Tools in Neurosciences. J. Neurosci..

[133] Assen A.H., Yassine O., Shekhah O., Eddaoudi M., Salama K.N. (2017). MOFs for the Sensitive Detection of Ammonia: Deployment of Fcu-MOF Thin Films as Effective Chemical Capacitive Sensors. ACS Sens..

[134] Daniel M., Mathew G., Anpo M., Neppolian B. (2022). MOF Based Electrochemical Sensors for the Detection of Physiologically Relevant Biomolecules: An Overview. Coord. Chem. Rev..

[135] Wang G.D., Li Y.Z., Shi W.J., Zhang B., Hou L., Wang Y.Y. (2021). A Robust Cluster-Based Eu-MOF as Multi-Functional Fluorescence Sensor for Detection of Antibiotics and Pesticides in Water. Sens. Actuators B Chem..

[136] Varadwaj A., Varadwaj P.R., Marques H.M., Yamashita K. (2018). Revealing Factors Influencing the Fluorine-Centered Non-Covalent Interactions in Some Fluorine-Substituted Molecular Complexes: Insights from First-Principles Studies. ChemPhysChem.

[137] Chen B., Yang Z., Qu X., Zheng S., Yin D., Fu H. (2021). Screening and Discrimination of Perfluoroalkyl Substances in Aqueous Solution Using a Luminescent Metal-Organic Framework Sensor Array. ACS Appl. Mater. Interfaces.

[138] Suwannakot P., Lisi F., Ahmed E., Liang K., Babarao R., Gooding J.J., Donald W.A. (2020). Metal-Organic Framework-Enhanced Solid-Phase Microextraction Mass Spectrometry for the Direct and Rapid Detection of Perfluorooctanoic Acid in Environmental Water Samples. Anal. Chem..

[139] Wang S., Niu H., Zeng T., Zhang X., Cao D., Cai Y. (2017). Rapid Determination of Small Molecule Pollutants Using Metal-Organic Frameworks as Adsorbent and Matrix of MALDI-TOF-MS. Microporous Mesoporous Mater..

[140] Li Y., Lu Y., Zhang X., Cao H., Huang Y. (2022). Cobalt-Embedded Nitrogen-Doped Carbon Nanosheets with Enhanced Oxidase-like Activity for Detecting Perfluorooctane Sulfonate. Microchem. J..

[141] Jia Y., Qian J., Pan B. (2021). Dual-Functionalized MIL-101(Cr) for the Selective Enrichment and Ultrasensitive Analysis of Trace Per- And Poly-Fluoroalkyl Substances. Anal. Chem..

[142] Tian Q., Sun M. (2019). Analysis of GenX and Other Per- and Polyfluoroalkyl Substances in Environmental Water Samples. Separation Science and Technology (New York).

[143] Wang Y., Darling S.B., Chen J. (2021). Selectivity of Per- and Polyfluoroalkyl Substance Sensors and Sorbents in Water. ACS Appl. Mater. Interfaces.

[144] Wang L., Pagett M., Zhang W. (2023). Molecularly imprinted polymer (MIP) based electrochemical sensors and their recent advances in health applications. Sens. Actuators Rep..

[145] Díaz-Álvarez M., Martin-Esteban A. (2021). Molecularly Imprinted Polymer-Quantum Dot Materials in Optical Sensors: An Overview of Their Synthesis and Applications. Biosensors.

[146] Bowei L., Qi J., Liu F., Zhao R., Arabi M., Ostovan A., Song J., Wang X., Zhang Z., Chen L. (2023). Molecular imprinting-based indirect fluorescence detection strategy implemented on paper chip for non-fluorescent microcystin. Nature.

